# Constituents of Essential Oil and Lipid Fraction from the Aerial Part of *Bupleurum scorzonerifolium* Willd. (Apiaceae) from Different Habitats

**DOI:** 10.3390/molecules23061496

**Published:** 2018-06-20

**Authors:** Zhargal Alexandrovich Tykheev, Svetlana Vasilievna Zhigzhitzhapova, Faqi Zhang, Vasiliy Vladimirovich Taraskin, Oleg Arnoldovich Anenkhonov, Larisa Dorzhievna Radnaeva, Shilong Chen

**Affiliations:** 1 Baikal Institute of Nature Management, Siberian Branch, Russian Academy of Sciences, Ulan-Ude 670047, Russia; gagarin199313@gmail.com (Z.A.T.); zhig2@yandex.ru (S.V.Z.); vvtaraskin@mail.ru (V.V.T.); radld@mail.ru (L.D.R.); 2 Key Laboratory of Adaption and Evolution of Plateau Biota, Northwest Institute of Plateau Biology, Chinese Academy of Sciences, Xining 810001, China; slchen@nwipb.cas.cn; 3 Qinghai Provincial Key Laboratory of Crop Molecular Breeding, Xining 810008, China; 4 Institute of General and Experimental Biology, Russian Academy of Sciences, Ulan-Ude, 670047, Russia; anen@yandex.ru

**Keywords:** *Bupleurum scorzonerifolium*, GC-MS, PCA, environmental

## Abstract

The essential oils and lipid fraction extracted from the aerial parts of *Bupleurum scorzonerifolium* were determined by a GC-MS method. In total, up to 67 components were identified. *cis*-*β*-Ocimene, *trans*-*β*-ocimene, limonene, *α*-pinene, *α*-copaene, *β*-elemene, and caryophyllene oxide were recognized as consistent components of the essential oil extracted from the aerial parts of *B. scorzonerifolium*, regardless of the habitat. The content of these components varied from traces to a significant amount. The volume of the lipid fraction varied from 2.73 to 9.38%. In total, 23 components were identified, including 20 fatty acids, two sterols, and one ketone. The major fatty acid components identified were 16:0, 18:2n9, and 18:1n9. The total content of these fatty acids reached up to 76.19%. The lipid fraction of the aerial parts of *B. scorzonerifolium* predominantly contained MUFA and PUFA, which confirmed the pharmacological value of the species. The main factors affecting the composition of essential oils and lipid fractions of *B. scorzonerifolium* are environmental ones that determine the moisture supply to the plants in semiarid and arid areas.

## 1. Introduction

As one of the most important and major components of traditional oriental medicine, *Bupleurum scorzonerifolium* Willd. is a perennial polycarpic herb growing up to 70 cm tall with compound umbels, which has an East Asian distribution area and is widespread in the eastern regions of Russia (Siberia and the Far East) as well as in Mongolia, China, and Korea [1]. The aerial parts of *B. scorzonerifolium* have been widely applied as a choleretic and hepatoprotective remedy in traditional Russian medicine [2]. The roots have been widely used in traditional Chinese medicine. *B. scorzonerifolium* and *B. chinense* are defined as the officinal plant of Chaihu—Bupleurum roots—in the Chinese Pharmacopeia [3]. Active compounds isolated from the roots, such as saikosaponins, polysaccharides, flavonoids, essential oils and fatty acids, possess various pharmacological functions including hepatoprotective, mild sedative, antipyretic, analgesic, anti-tussive, immunomodulatory effect, anti-fibrotic, anti-liver cancer as well as promoting liver regeneration [4,5,6].

Previous studies have been dedicated to the chemical constituents of essential oils of the aerial parts of *B. scorzonerifolium* growing within Russia, in particular, the Krasnoyarsk region [7,8], Irkutsk region [9], and Mongolia [10]. One study also provided detailed information about the essential oils extracted from the roots of plants growing in China [11]. The fatty acid constituents of *B. scorzonerifolium*’s roots from Russia (Republic of Buryatia) and Mongolia, as well as *B. chinense* from China, have been reported in [12,13]. Additionally, the chemical composition of lipids from ten *Bupleurum* species has been given in a range of papers from Turkey [14,15] and the leaf and seeds of *B. lancifolium* from Iran [16]. However, studies of essential oil composition were generally incomplete and related to plants growing in scattered populations. Furthermore, the lipid fraction of the aerial part of *B. scorzonerifolium* has yet to be studied. The lipid fraction constituents for *Bupleurum scorzonerifolium* Willd., the species growing within Russia (Republic of Buryatia, Trans–Baikal territory), and Mongolia, are reported in this study for the first time. In this study, we investigated and conducted a comparative analysis of the essential oils and lipid fraction of the aerial parts of *B. scorzonerifolium* from different habitats and tried to establish some possible relationships between the chemical composition and environmental differences.

## 2. Results and Discussion

### 2.1. Essential Oil Composition

The extracted essential oils were light green to light yellow, and the volume was about 0.18% to 1.66% (*v*/*w*). Using the GC-MS technique 67 components were identified in the essential oil of *B. scorzonerifolium*, including acyclic, aromatic, mono- and sesquiterpene compounds (Table 1). General components identified in samples from the Republic of Buryatia (Russia) were monoterpenes: *β*-myrcene (2.65–14.25%), *cis*-*β*-ocimene (0.62–18.56%), *trans*-*β*-ocimene (2.10–14.79%), limonene (2.06–10.78%), *γ*-terpinene (0.49–5.66%), *α*-pinene (0.47–4.67%), sabinene (0.93–4.80%), *β*-pinene (0.44–3.58%); sesquiterpenes: *β*-elemene (0.48–4.04%), humulene (0.89–2.91%), germacrene D (9.50–41.81%), caryophyllene (3.57–11.35%), bicyclogermacrene (1.13–3.56%), *δ*-cadinene (0.47–4.76%), caryophyllene oxide (0.60–6.87%), spathulenol (0.54–6.55%), and the aromatic compound *p*-cymol (0.41–21.73%). The main components of samples from the Trans–Baikal region (Russia) were monoterpenes: *β*-myrcene (3.50%), limonene (6.31%), *α*-pinene (5.86%); sesquiterpenes: germacrene D (5.13%), caryophyllene (2.49%), *γ*-muurolene (3.22%), mint oxide (4.12%), *α*-cadinol (2.50%), spathulenol (10.36%); aromatic compounds like *p*-cymol (12.74%); and an aldehyde, myristylaldehyde (8.86%).

Components of essential oils extracted from plants originating from Mongolia were monoterpenes: *β*-myrcene (0.62–8.35%), *cis*-*β*-ocimene (0.37–9.22%), *trans*-*β*-ocimene (0.49–3.08%), limonene (2.64–7.08%), *γ*-terpinene (0.09–2.53%), *α*-pinene (0.11–5.62%), *β*-pinene (0.92–8.09%); sesquiterpenes: *β*-elemene (1.16–3.68%), humulene (1.71–14.66%), germacrene D (15.56–41.34%), caryophyllene (3.91–12.83%), bicyclogermacrene (3.53–7.61%), caryophyllene oxide (0.71–7.30%), spathulenol (0.63–7.42%); and aromatic compounds: *p*-cymol (0.18–10.56%). Comparison with data from the literature [7,8,9,10] showed that *cis*-*β*-ocimene, *trans*-*β*-ocimene, limonene, *α*-pinene, *α*-copaene, *β*-elemene, and caryophyllene oxide were consistent components in the essential oil of *B. scorzonerifolium*’s aerial parts, regardless of habitat. The contents of these components varied from traces to a significant amount.

Humulene was a specific component of the essential oil of plants originating from the Republic of Buryatia, Trans–Baikal region, and Mongolia. At the same time, pinocarvone, *p*-cymen-8-ol, and *α*-campholenal were specific only for samples from the Trans–Baikal region. Samples from the Krasnoyarsk region were characterized by an accumulation of trace amounts of compounds such as geranyl acetate, *γ*-bisabolene, *iso*-elemicin, *epi*-*α*-bisabolol, *iso*-caryophyllene, *t*-cadinol, *δ*-cadinol, *iso*-spathulenol, benzyl alcohol, asarone, *n*-octyl butanoate, dihydrocitronellol, linalyl butanoate, salicylaldehyde, and pentadecanal [7,8]. Monoterpenes: *α*-fellandrene, (1*S*,5*S*)-2(10)-pinene, *δ*-3-carene, *p*-mentha-1,3,8-diene-6-ol; and sesquiterpenes: *γ*-elemene, seychellene, *β*-guaiene, *γ*-gurjunene, *γ*-himachalene, naphthalene,1,2,4a,5,8,8a-hexahydro-4,7-dimethyl-1-(1-methylethyl)-[1*S*-(1*α*,4a*β*,8a*α*)]-, 9-methoxy-calamene, aromadendrene oxide-(2), *iso*-aromadendrene epoxide, muurolan-3,9(11)-dien-10-peroxide, and ledene oxide were specific for samples from the Irkutsk region [9].

**Figure 1:**
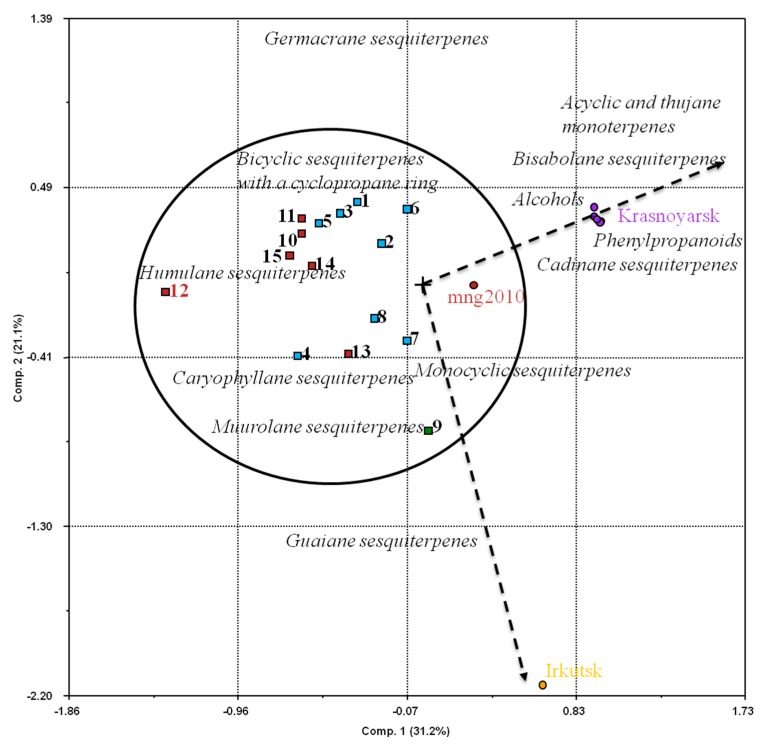
Principal component analysis biplot of essential oil components from *B. scorzonerifolium* samples collected from different regions. Numerals indicate own data (Table 1). Light-blue-filled quadrants indicate samples from Buryatia (Russia), green-filled quadrant is the sample from the Trans–Baikal region (Russia), brown-filled quadrants are the samples from Khentii aimag (Mongolia). Brown-filled circle (mng2010) is according to [10]. “Irkutsk” indicates the *B. scorzonerifolium* sample from the Irkutsk region (Russia) according to [9]. “Krasnoyarsk” indicates the *B. scorzonerifolium* sample from the Krasnoyarsk region (Russia) according to [7,8].

PCA was also used to evaluate the variation among *B. scorzonerifolium* samples from different habitats based on the structural groups classification of terpenes by Semenov [18]. The first choice for most data is an orthogonal projection on the plane of the two principal components (PC1, PC2, or PC3). The projection plane is essentially a planar two-dimensional screen positioned such that the data are displayed with the smallest distortions. Despite that, PC1 and PC2 explained a little over 50% of the total variance as they were chosen from three extracted combinations. In particular, these components contained specific variables which showed that the samples were divided into groups according to the geographical location with Lake Baikal as a natural boundary between them (Figure 1). For instance, the Republic of Buryatia, Trans–Baikal region, and Mongolia are located to the east side of Lake Baikal, while the Irkutsk and Krasnoyarsk regions are to the west. The climate of Siberia and its cross-border Mongolia is sharply continental. The great extent and great difference in the geography determine the variability of climates of individual regions of this territory. For instance, climatic differences between the western and eastern regions of Siberia are prominent. The climate of Eastern Siberia (Irkutsk region, Buryatia region, and Trans–Baikal region) is generally more continental, while the territory of Western Siberia (including the Krasnoyarsk region) is milder and has more precipitation. The main reason for these differences is that wet air masses from the Atlantic Ocean become increasingly weaker in the course of transferring through Europe and Siberia. As a result, they only have residual influence on the climate in the Irkutsk region, and almost do not affect the climate of areas in the Republic of Buryatia located to the east of the mountains that surround Lake Baikal. Due to this, a large part of the Buryatia region is characterized by a semiarid climate. At the same time, the huge water body of Lake Baikal markedly modifies climatic conditions on the coastal areas, significantly smoothing out diurnal temperature changes and even seasonal ones. To the east and south from Buryatia, namely, in the Trans–Baikal region and Mongolia, the climate is more continental and arid.

The location of the samples projection and the content of sesquiterpenes of the biogenetic germacrane similarity (germacrane and guaiane types of sesquiterpenes) on the opposite sides of the biplot showed the absence of germacrene type sesquiterpenes in samples from the Irkutsk region, while the guaiane type of sesquiterpenes were not found in samples from the Republic of Buryatia, Trans–Baikal region, Krasnoyarsk region, and Mongolia.

The samples from the most continental territories (namely, the Republic of Buryatia, Trans–Baikal territory and Mongolia) of semiarid and arid regions of Asia were grouped in a single cluster on the biplot. These populations were characterized by high contents of humulane, caryophyllane, muurolane types of sesquiterpenes, monocyclic sesquiterpenes, and bicyclic sesquiterpenes with a cyclopropane ring. Samples from territories with a higher level of precipitation and more temperate conditions (Irkutsk and Krasnoyarsk regions) were grouped on the right side of the biplot. The samples from Krasnoyarsk were focused in the upper side of the chart. These samples were characterized by high levels of cadinane and bisabolane types of sesquiterpenes, alcohols, phenylpropanoids, acyclic and thujane monoterpenes. Samples from the Irkutsk region were located in the lower part and predominantly contained the guaiane type of sesquiterpenes. Thus, we could assume that *B. scorzonerifolium*’s essential oil composition is influenced by environmental factors that determine the moisture conditions in sites inhabited by *Bupleurum* plants.

### 2.2. Lipid Fraction Composition

The extracted lipid fractions were solid, viscous, had a dark green color and a pleasant smell. The volume varied from 2.73 to 9.38% being non-identical in the different habitats of plants (Table 1). The composition of lipids was determined by GC-MS. In total, 23 components were identified in *B. scorzonerifolium*’s lipid fraction including 20 fatty acids, two sterols, and one ketone (Table 2). The major fatty acids identified in the lipid fraction from the aerial parts of *B. scorzonerifolium* were 16:0, 18:2n9, and 18:1n9. The total content of the above-mentioned fatty acids reached up to 76.19% (sample No. 14). They were also dominant in the aerial part of other *Bupleurum* species from Turkey [14,15] as well as the roots of *B. scorzonerifolium* from Russia (Republic of Buryatia), Mongolia, and *B. chinense* from China [12,13]. The content of polyunsaturated fatty acids (PUFA) ranged from 24.02 to 36.69% of the total lipid fraction. The dominant PUFA was diene acid 18:2n9 (20.17–31.95%). Fatty acid 16:3n7 (up to 4.63%) was found in a significant amount, while 18:3n6 and 20:3n8 (up to 2%) were minor. The content of monounsaturated fatty acids (MUFA) was in the range of 18.60–33.64%. The dominant MUFA was *cis*18:1n9, with the highest percentage in sample No. 5 (30.18%) and the lowest percentage was No. 7 (15.11). The content of 16:1n9 was around 0.64–2.32%. *trans*18:1n9 and 20:1n1 were found in minor amounts.

There were 12 saturated fatty acids (SFA) in the lipid fraction. Their total content ranged from 22.26% to 40.40%. The dominant SFA was 16:0 (13.03–29.25%) while 14:0 (1.15–3.95%), 18:0 (1.67–3.16%), 20:0 (0.65–2.12%), and 22:0 (0.97–2.62%) were found in significant quantities and 12:0, nonanedioic acid, 15:0, 17:0, 20:0, 23:0, 24:0, and 26:0 were minor. The studied samples contained such sterols as stigmasterol (1.37–3.43%) and *β*-sitosterol (1.58–3.71%). The content of 10-nonadecanone for most of the samples was significant (3.23–11.28%), however, in sample No. 2, the amount was minimal at 0.09%. 

**Figure 2:**
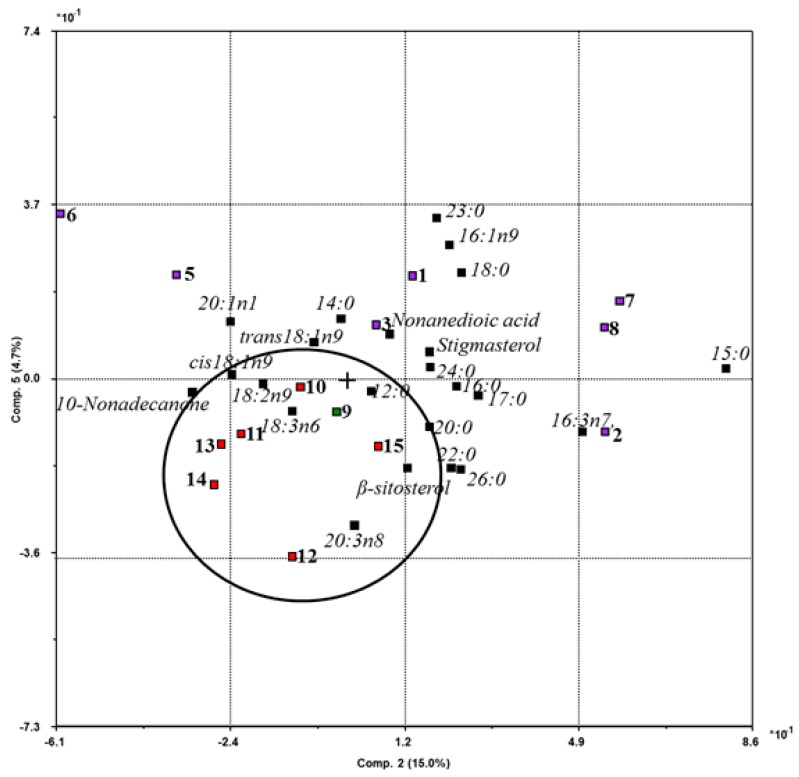
The principal component analysis biplot of the lipid fraction extracted from *B. scorzonerifolium* samples collected from different regions. Numerals indicate our data (Table 3). Violet-filled quadrants indicate samples from Buryatia (Russia), green-filled quadrants are samples from the Trans–Baikal region (Russia), red-filled quadrants are the samples from Khentii aimag (Mongolia).

PCA of the lipid fraction of plants from different habitats showed that the samples from more arid steppe territories (Trans–Baikal region and Mongolia) were grouped into a single cluster on the biplot (Figure 2) and were characterized by a high level of unsaturated fatty acids. According to the obtained results, the lipid fraction of the aerial part of *B. scorzonerifolium* predominantly contained MUFA and PUFA, which confirmed the pharmacological value of the species. Applying unsaturated fatty acids can alleviate the symptoms of some chronic and degenerative (cardiovascular and inflammatory) diseases [19].

## 3. Materials and Methods

### 3.1. Plant Materials

Plant materials were collected from June 2014 to August 2017 in Russia (Republic of Buryatia, Trans–Baikal territory) and Mongolia (Khentii aimag; Table 3). All samples were collected during the flowering period and air-dried before being ground into a fine powder. Voucher specimens were identified by Dr. Oleg A. Anenkhonov from the Institute of General and Experimental Biology, Siberian Branch, Russian Academy of Sciences (IGEB SB RAS). Some of the samples were deposited into the Herbarium of IGEB SB RAS (UUH; for voucher numbers see Table 3), another one was stored in the local collection of the laboratory for the Chemistry of Natural Systems (Baikal Institute of Nature Management, Siberian Branch, Russian Academy of Sciences; under mark TZA).

### 3.2. Isolation of Essential Oils

A portion of the powdered plant material (30~40 g) was extracted by hydrodistillation for 3 h in a Clevenger-type collector apparatus. Essential oils were isolated following the method described in the Pharmacopoeia of Russia [17]. The essential oils produced in this study were extracted from all of the aerial parts of the plant. All experiments were done in triplicate and the obtained results were expressed on the basis of air-dry weight.

### 3.3. GC-MS Analysis of Essential Oils

Gas chromatography-mass spectrometry (GC-MS) analysis was carried out to determine the composition of the essential oils using an Agilent 6890 gas chromatograph (Agilent Technologies, Santa Clara, CA, USA) equipped with an HP 5973 quadrupole mass selective detector (Hewlett-Packard, Palo Alto, CA, USA) and an HP-5MS capillary column (30 m × 0.25 mm × 0.2 μm; Hewlett-Packard). MS was carried out with the electron impact at 70 eV and the electron multiplier set at 2200 V. Helium was used as the carrier gas at a flow rate of 1 mL/min. The column temperature was set from 50 to 240 °C. The oven temperature was programmed to increase from 50 to 240 °C, increasing at a rate of 4 °C/min with an initial hold for 5 min, and then from 240 to 280 °C, increasing at a rate of 20 °C/min with a final hold for 5 min. The injector and detector temperatures were 280 and 250 °C, respectively. The purity of the helium gas was 99.999%. The column pressure was set to 52.8 × 10^3^ Pa. The split ratio was 60:1.

### 3.4. Essential Oil Compound Identification

Essential oil constituents were identified by comparing their mass spectra with standards from the National Institute of Standards and Technology [20] and with calculated linear retention indices (RIs) in the literature [21]. The RIs were obtained by co-injection with a mixture of linear C_8_–C_20_ hydrocarbons (Sigma, St. Louis, MO, USA). Identification was approved when computer matching with the mass spectral libraries had a probability above 90%. The contents of the oil components were identified by the relative amount (%) of the individual oil components and were expressed as a percentage peak area relative to the total peak area based on the GС-MS analyses of the oils.

### 3.5. Isolation of Lipids

The lipid fraction was isolated by a modified Bligh and Dyer method [22]. A 30~40 g portion of the powdered plant material were extracted with 300 mL of mixture chloroform/methanol (1/2, *v*/*v*) homogenizer IKA T18 ULTRA-TURRAX T25 (IKA Werke GmbH., Staufen, Germany) for 5 min. Then, the homogenate was filtered, and the plant residues on the filter were again extracted with a mixture of 300 mL of chloroform/methanol (1/2, *v*/*v*) and 80 mL of distilled water. The homogenate was filtered and plant residues on the filter were wet out with a 150 mL mixture of chloroform/methanol (1/2, *v*/*v*). A total of 25 mL of chloroform and 29 mL of distilled water were added to the total extract. Chloroform and water–methanol layers were divided with a separation funnel. The chloroform layer was evaporated under reduced pressure in an IR-50LT rotary evaporator (Labtex, Moscow, Russia) and the extracted lipid fraction was weighed. All experiments were done in triplicate and the results were expressed on the basis of air-dry weight.

### 3.6. Methylation of Lipids

A total of 200–300 mg of lipid fraction was mixed with 2 mL 2 M hydrochloric acid in methanol in vial with a screw cap, saponified by heating in beaker at 90 °C for 2 h. Then, the mixture after cooling was dried under an argon flow to 0.5 mL. One mL of distilled water was added and extracted thrice with 0.5 hexane. The combined hexane fraction was analyzed by GC-MS.

### 3.7. GC-MS Analysis of Derivatives from the Lipid Fraction

In order to determine the composition of the lipid fraction, gas chromatography-mass spectrometry (GC-MS) analysis was performed using an Agilent 7890B gas chromatograph with a 7000C triple quadrupole mass spectrometer as the detector and an HP-5MS capillary column (30 m × 0.25 mm × 0.2 µm; Hewlett-Packard). Helium (99.999% purity) was used as the carrier gas at a flow rate 1.5 mL/min. The oven temperature was programmed as follows: it was kept at a constant of 125 °С for 0.5 min, from 125 °C to 320 °C (at rate of 7 °C/min), and kept constant at 320 °C for 0.5 min. The injector and detector temperatures were set to 280 °C and 250 °C, respectively. The split ratio was adjusted to 40:1. The MS data were acquired in scanning mode at a speed of 2.5 s per scan.

### 3.8. Lipid Fraction Compounds Identification

The percent composition of the lipid fraction derivatives was calculated from the GC peak areas relative to the total peak area based on the GС-MS analyses of the lipid fraction. Qualitative analysis was based on comparing the retention times and total mass spectra of the corresponding pure compounds using NIST14.L and standard mixtures of Bacterial Acid Methyl Esters (CP Mix, Supelco, Bellefonte, PA, USA) and Fatty Acid Methyl Esters (Supelco, 37 compounds, FAME Mix, 10 mg/mL in CH_2_Cl_2_). Iso-, antheiso, 2-OH- fatty acids are indicative of bacteria and microscopic fungi and can often be found among plant fatty acids (database https://plantfadb.org/). Thus, due to the fact that the composition of the lipid fraction of aerial part of *B. scorzonerifolium* was investigated for the first time, we also used the BAME standard with the FAME standard.

### 3.9. Statistical Analysis

The chemical composition data presented in Table 1 and Table 2 were subjected to multivariate statistical analysis using principal component analysis (PCA). All statistical analysis were conducted using the Sirius software ver. 6.0 (Pattern Recognition Systems, Bergen, NORWAY) [23]. Compounds found in all or the majority of the samples were subjected to statistical analysis, and their relative values (i.e., percentage of the sum) were logarithmically transformed. This process allowed us to derive an equation that could be used to define quantitative differences among individual compounds. As a scaling method, the unit or unit variance scaling was applied, which is common and uses the standard deviation as the scaling factor [24,25]. The model was leave one out cross validated. The standard deviation from the average values of the amounts of lipid fraction components was calculated using the Excel 2013 software (Microsoft, Redmond, WA, USA) package.

## 4. Conclusions

This work investigated the chemical composition of the essential oils and lipid fraction of *B. scorzonerifolium* from different habitats. Compared with previous studies, *cis*-*β*-ocimene, *trans*-*β*-ocimene, limonene, *α*-pinene, *α*-copaene, *β*-elemene, caryophyllene oxide were consistent components of the aerial parts of *B. scorzonerifolium,* regardless of habitat. Their content varied from traces to significant amounts. It was shown that the aridity of the area facilitated a higher content of the humulane, caryophullane, and muurolane types of sesquiterpenes, monocyclic sesquiterpenes, and bicyclic sesquiterpenes with a cyclopropane ring. For this reason, intensifying biosynthesis could be assumed as a probable underlying mechanism of this relation. For the first time, the chemical constituents of the lipid fraction extracted from the aerial part of *B. scorzonerifolium* from the Republic of Buryatia, Trans–Baikal region, and Mongolia were investigated by GC-MS. 16:0, 18:2n9 and 18:1n9 were identified as the major fatty acids. According to the results, the lipid fraction of the aerial parts of *B. scorzonerifolium* predominantly contained MUFA and PUFA, which confirmed the pharmacological value of the species. It is probable that the main factors affecting the composition of essential oils and lipid fractions of *B. scorzonerofolium* are environmental ones that determine the moisture conditions at the sites inhabited by *Bupleurum* plants. To verify this assumption and for a better understanding of the compounds’ synthesis, studies of other *Bupleurum* species from semiarid and arid territories of Asia should be conducted in the future.

## Figures and Tables

**Table 1:** Chemical composition of essential oils extracted from the aerial parts of *Bupleurum scorzonerifolium* Willd. from different countries or regions.

RI ^a^		Compound	Peak Area % (Percentage) ^b^														
Exp.	Lit. [17]		1	2	3	4	5	6	7	8	9	10	11	12	13	14	15
	ΣAldehydes:		0.53	0.68	0.08	0.60	2.86	1.69		1.71	13.31	6.90	0.19	1.12	2.65	3.23	3.14
806	806	Hexanal		0.17	0.08	0.60					1.41	0.12					
958	958	Benzaldehyde									0.38						
1241	1241	Cumin aldehyde									0.38						
1317	1317	(2*E*,4*E*)-Deca-2,4-dienal						0.15		0.22	1.03	0.15	0.06				
1409	1409	Dodecanal						0.26		1.49	1.25	0.32	0.07		1.78		1.31
1601	1601	Myristylaldehyde	0.53	0.51							8.86	2.26		1.12			1.83
1817	1817	Hexadecanal					2.86	1.28				4.05	0.06		0.87	3.23	
	ΣAromatics:		0.47	1.59	0.99	0.41		5.31	21.73	20.38	12.76	1.62	10.61	0.18	7.83	6.26	2.05
1024	1024	*p*-Cymol	0.47	1.59	0.99	0.41		5.31	21.73	20.38	12.76	1.50	10.56	0.18	7.83	6.26	2.05
1406	1406	Methyl eugenol										0.12	0.05				
	ΣAliphatic hydrocarbons:			0.28	0.08	0.00	0.28	0.07		0.43	1.03	2.47	2.69	15.37	4.08		
900	900	*n*-Nonane											0.08	0.48	0.07		
1100	1100	*n*-Undecane		0.28	0.08		0.28				0.70	1.85	1.42	13.75	2.96		
1300	1300	*n*-Tridecane						0.07		0.43	0.33	0.40	1.13	1.14	0.67		
2300	2300	*n*-Tricosane										0.22	0.06		0.38		
	ΣAcyclic monoterpenes:		33.17	32.44	28.15	4.91	15.98	28.89	20.29	21.22	5.83	14.35	15.28	6.83	11.97	17.13	18.43
991	991	*β*-Myrcene	12.04	13.76	10.89	2.65	3.56	14.25	13.77	13.10	3.50	5.29	0.62	3.02	8.52	7.98	8.35
1038	1038	*cis*-*β*-Ocimene	18.56	16.58	2.47	2.26	1.13	2.04	0.62	1.09	1.55	7.71	12.33	3.32	0.37	8.54	9.22
1048	1048	*trans*-*β*-Ocimene	2.57	2.10	14.79		11.29	12.60	5.90	7.03	0.40	1.35	2.33	0.49	3.08	0.61	0.86
1162	1162	Pinocarvone									0.38						
	ΣMonocyclic monoterpenes:		7.90	10.00	9.18	2.89	3.57	17.88	12.82	14.26	17.00	8.28	12.94	6.23	10.74	8.41	7.29
1017	1017	*α*-Terpinene			0.13			0.29									
1028	1028	Limonene	6.22	8.41	6.14	2.89	2.06	10.78	9.69	7.68	6.31	4.38	2.64	6.23	7.08	6.1	5.63
1031	1031	1,8-Cineol										0.09					
1058	1058	*γ*-Terpinene	1.36	1.2	2.30		0.49	5.42	2.47	5.66	1.27	2.53	0.09		1.63	2.31	1.66
1088	1088	Terpinolene			0.10			0.21					4.48				
1089	1089	*p*-Cymene									0.51						
1093	1093	Rose furan						0.19		0.11	0.70				0.11		
1112	1112	Mentha-1,3,8-triene						0.53					4.89				
1177	1177	Terpinen-4-ol	0.32	0.39	0.44		0.89	0.06	0.23	0.37	0.76	0.26	0.24		0.45		
1186	1186	*p*-Cymen-8-ol									1.25						
1191	1191	*α*-Terpineol			0.07		0.13	0.09			0.54		0.03		0.16		
1199	1199	Methyl Chavicol											0.46				
1210	1210	Verbenone							0.43	0.44	0.65		0.11				
1219	1219	*trans*-Carveol						0.09			1.17				0.46		
1233	1233	*cis*-Carveol									0.63				0.15		
1245	1245	Carvone									0.95						
1367	1367	*cis*-Carveol acetate						0.22			1.47				0.70		
1488	1488	*β*-Ionone									0.79	1.02					
	ΣBicyclic monoterpenes:		4.17	10.93	4.73	1.37	2.75	8.69	7.83	10.34	10.93	3.93	15.31	5.60	10.10	2.65	7.01
926	926	*β*-Thujene		0.15	0.16			0.79			0.24	0.10	1.55		0.33		
932	932	*α*-Pinene	1.04	4.46	0.84		0.47	1.25	1.89	4.67	5.86	1.17	0.11	3.66	2.72	1.73	5.62
947	947	Camphene		0.29	0.14			0.29	0.63	0.27	0.38		1.29		0.37		
973	973	Sabinene	1.75	4.08	2.40	0.93	1.66	4.80	2.13	1.82	0.33	0.89	3.94		0.61		0.25
975	975	*β*-Pinene	1.38	1.95	1.12	0.44	0.47	1.24	2.48	3.58	1.60	1.51	8.09	1.94	3.13	0.92	1.14
1144	1144	Camphor						0.17			0.84	0.14			2.32		
1197	1197	Myrtenol									0.92				0.36		
1287	1287	Bornyl acetate			0.07		0.15	0.15	0.70		0.76	0.12	0.33		0.26		
	ΣAliphatic sesquiterpenes:		1.11	0.13	1.72		1.44	2.48	0.64	0.93	0.81	3.77	1.38	0.65	1.22		1.18
1458	1458	*β*-Farnesene	0.70		0.35		0.36	0.80				0.52	0.24		0.24		
1496	1496	(*Z*,*E*)-*α*-Farnesene	0.41	0.13								1.20					1.18
1510	1510	(*E*,*E*)-*α*-Farnesene			1.37		1.08	1.68	0.64	0.93	0.81	2.05	1.14	0.65	0.98		
	ΣMonocyclic sesquiterpenes:		39.51	30.56	37.15	47.46	50.72	24.10	15.96	13.17	10.55	41.04	23.79	41.94	21.25	45.17	32.77
1126	1126	*α*-Campholenal									0.55						
1378	1378	*α*-Copaene	0.39	0.51	0.82	1.98	0.56	0.40	0.27	0.41	1.03	0.62	0.30	0.33	0.40		0.89
1392	1392	*β*-Elemene	0.50	1.85	0.48	4.04	4.73	3.08	2.00	1.59	1.14	3.68	1.16	2.21	2.31	1.29	1.20
1432	1432	*β*-Copaene	0.57		0.81		0.75	0.47				0.71	0.38		0.25		0.32
1445	1445	*iso*-Germacrene D			0.33	0.59						0.26					0.16
1456	1456	Humulene	1.23	0.89	1.45	2.91	1.83	1.69	2.26	1.67	1.65	2.63	5.72	14.66	1.71	2.54	3.19
1484	1484	Germacrene D	36.82	26.76	32.01	35.68	41.81	17.94	11.43	9.50	5.13	31.57	15.76	24.74	15.56	41.34	25.71
1688	1688	Germacra-4(15),5,10(14)-trien-1-ol		0.55	1.25	2.26	1.04	0.52			1.05	1.57	0.47		1.02		1.30
		ΣBicyclic sesquiterpenes:	12.60	12.10	17.28	35.81	21.20	10.02	16.29	14.56	17.42	16.60	16.88	21.45	22.74	17.15	27.00
1339	1339	Bicycloelemene	0.34		0.47		0.52	0.23				0.56	0.63	0.36	0.29		
1392	1392	*β*-Cubebene			2.60			0.43									2.43
1421	1421	*β*-Cedrene															0.29
1422	1422	Caryophyllene	5.60	5.08	5.85	11.35	9.60	4.30	3.57	5.58	2.49	7.35	8.09	10.65	3.91	12.83	8.31
1480	1480	*γ*-Muurolene		0.78	1.39	5.13			1.46	1.39	3.22			1.43	2.22	1.85	1.63
	1500	Bicyclogermacrene	2.69	1.63	3.46	3.56	4.88	1.81	1.22	1.13	0.48	3.57	6.04	5.23	3.53		7.61
1507	1507	Cuparene	0.67	0.30		1.01					1.08						1.46
1517	1517	*γ*-Cadinene			0.30		0.32	0.17				0.29	0.10	0.17	0.37		
1527	1527	*δ*-Cadinene	1.21	0.73	1.37	4.76	1.64	0.94	0.47	0.62	1.14	1.47	0.64	1.10	1.03	1.33	2.01
1565	1565	*β*-Calacorene		0.13		0.40		0.06			0.63	0.22	0.07				
1568	1568	Mint oxide		1.05	0.28		0.36	0.38	2.70		4.12	0.31			2.92		
1586	1586	Caryophyllene oxide	0.60	1.53		5.18	1.55	0.98	6.87	5.84	0.86	0.88	0.71	1.15	7.30	1.14	2.18
1598	1598	Mint ketone			0.11						0.90	0.19	0.13				
1644	1644	*t*-muurolol	0.57	0.32	1.45	4.42	0.77					0.60	0.14	0.73			
1658	1658	*α*-Cadinol	0.92	0.55			1.56	0.72			2.50	1.16	0.33	0.63	1.17		1.08
	ΣTricyclic sesquiterpenes:		0.54	1.29	0.64	6.55	1.20	0.87	4.44	3.00	10.36	1.04	0.93	0.63	7.42		1.13
1580	1580	Spathulenol	0.54	1.29	0.64	6.55	1.20	0.87	4.44	3.00	10.36	1.04	0.93	0.63	7.42		1.13

^a^ RI, retention indices: experimental, for our data (RI, retention index as determined on a HP-5MS column using the homologous series of n-hydrocarbons). ^b^ Percentage values are means of three determinations with an RSD% for the main components below 5% in all cases.

**Table 2:** Chemical composition of fatty acids extracted from the aerial parts of *Bupleurum scorzonerifolium* Willd. from different countries or regions (as methyl esters).

RT ^c^	Compound	Peak Area % (Percentage)													
		1	2	3	5	6	7	8	9	10	11	12	13	14	15
7.35	12:0	1.43 ± 0.05	0.29 ± 0.00	1.19 ± 0.02	0.48 ± 0.02	0.46 ± 0.02	0.73 ± 0.05	0.85 ± 0.05	1.26 ± 0.07	1.49 ± 0.02	0.56 ± 0.02	0.88 ± 0.05	0.46 ± 0.02	0.89 ± 0.02	1.05 ± 0.05
7.79	Nonanedioic acid	1.52 ± 0.07	0.71 ± 0.02	0.81 ± 0.05	0.38 ± 0.02	0.56 ± 0.02	0.55 ± 0.02	0.55 ± 0.02	1.15 ± 0.05	0.86 ± 0.02	0.86 ± 0.02	0.28 ± 0.02	0.44 ± 0.02	0.94 ± 0.05	0.89 ± 0.05
11.25	14:0	2.22 ± 0.05	1.15 ± 0.02	3.95 ± 0.15	1.61 ± 0.07	2.47 ± 0.05	2.39 ± 0.10	2.54 ± 0.05	3.22 ± 0.07	2.74 ± 0.10	1.48 ± 0.05	2.03 ± 0.10	1.69 ± 0.05	2.26 ± 0.02	2.44 ± 0.10
13.26	15:0	0.75 ± 0.02	0.68 ± 0.02	0.70 ± 0.02	0.29 ± 0.00	0.22 ± 0.00	1.88 ± 0.07	1.36 ± 0.05	0.62 ± 0.02	0.77 ± 0.02	0.49 ± 0.02	0.53 ± 0.02	0.38 ± 0.02	0.34 ± 0.02	0.85 ± 0.02
15.29	16:0	25.65 ± 1.24	29.25 ± 0.45	20.02 ± 0.32	14.11 ± 0.62	15.77 ± 0.70	21.56 ± 0.77	19.51 ± 0.94	23.17 ± 1.04	19.82 ± 0.70	13.03 ± 0.40	18.99 ± 0.92	14.75 ± 0.35	20.19 ± 0.65	19.25 ± 0.84
17.23	17:0	0.74 ± 0.02	0.86 ± 0.02	0.75 ± 0.02	0.37 ± 0.02	0.42 ± 0.02	0.75 ± 0.02	0.73 ± 0.02	0.50 ± 0.02	0.43 ± 0.02	0.78 ± 0.02	0.43 ± 0.02	0.76 ± 0.02	0.55 ± 0.02	0.69 ± 0.02
19.12	18:0	2.96 ± 0.15	2.62 ± 0.07	3.16 ± 0.07	2.07 ± 0.10	2.39 ± 0.10	3.55 ± 0.15	3.04 ± 0.10	2.80 ± 0.12	2.53 ± 0.12	1.86 ± 0.07	1.71 ± 0.07	2.00 ± 0.10	1.67 ± 0.07	2.58 ± 0.12
22.74	20:0	0.87 ± 0.02	0.92 ± 0.02	1.14 ± 0.05	0.65 ± 0.02	0.81 ± 0.02	1.61 ± 0.05	1.92 ± 0.07	1.06 ± 0.05	1.00 ± 0.05	1.98 ± 0.10	0.90 ± 0.02	2.12 ± 0.07	0.95 ± 0.02	1.15 ± 0.05
26.02	22:0	1.42 ± 0.05	1.13 ± 0.05	1.26 ± 0.05	0.98 ± 0.05	1.36 ± 0.07	2.12 ± 0.10	2.62 ± 0.12	2.03 ± 0.10	1.92 ± 0.07	1.09 ± 0.05	4.91 ± 0.20	0.97 ± 0.05	1.04 ± 0.05	1.75 ± 0.07
27.59	23:0	0.70 ± 002	0.28 ± 0.00	0.70 ± 0.02	0.41 ± 0.00	0.69 ± 0.02	0.88 ± 0.02	1.01 ± 0.05	0.57 ± 0.02	0.71 ± 0.02	0.35 ± 0.00	0.46 ± 0.00	0.37 ± 0.00	0.33 ± 0.00	0.68 ± 0.02
29.10	24:0	1.43 ± 0.05	0.56 ± 0.02	1.25 ± 0.05	0.56 ± 0.02	1.18 ± 005	1.57 ± 0.07	1.96 ± 0.05	1.70 ± 0.07	1.40 ± 0.05	0.77 ± 0.02	1.68 ± 0.05	0.79 ± 0.02	0.93 ± 0.02	1.50 ± 0.05
31.98	26:0	0.71 ± 0.02	0.74 ± 0.02	0.50 ± 0.02	0.33 ± 0.00	0.43 ± 0.02	0.66 ± 0.02	1.08 ± 0.05	1.14 ± 0.05	0.67 ± 0.02	0.94 ± 0.02	0.56 ± 0.02	0.57 ± 0.02	0.83 ± 0.02	0.68 ± 0.02
ΣSFA		40.40 ± 1.86	39.19 ± 1.24	35.42 ± 1.17	22.26 ± 0.97	26.75 ± 1.34	38.26 ± 0.57	37.15 ± 0.74	39.23 ± 0.84	34.35 ± 1.29	24.18 ± 0.57	33.35 ± 1.34	25.30 ± 1.19	30.91 ± 0.82	33.50 ± 0.94
14.87	16:1n9	1.49 ± 0.07	2.32 ± 0.10	1.04 ± 0.05	1.50 ± 0.07	1.54 ± 0.07	2.11 ± 0.10	1.80 ± 0.07	0.84 ± 0.02	0.64 ± 0.02	2.03 ± 0.10	0.80 ± 0.02	2.04 ± 0.10	0.71 ± 0.02	0.77 ± 3.22
18.68	cis18:1n9	20.03 ± 0.60	20.64 ± 1.02	23.31 ± 1.04	30.18 ± 1.07	26.29 ± 1.14	15.11 ± 0.70	16.09 ± 0.77	22.38 ± 0.72	25.53 ± 0.99	24.20 ± 0.97	20.62 ± 0.94	21.87 ± 0.72	24.06 ± 0.82	24.22 ± 1.12
18.74	trans18:1n9	0.87 ± 0.02	0.71 ± 0.02	0.98 ± 0.05	0.97 ± 0.05	0.94 ± 0.02	0.70 ± 0.02	1.15 ± 0.05	0.69 ± 0.02	1.04 ± 0.05	0.93 ± 0.02	0.74 ± 0.02	0.97 ± 0.02	0.84 ± 0.02	0.97 ± 0.02
22.27	20:1n1	1.05 ± 0.05	0.56 ± 0.02	0.71 ± 0.02	0.99 ± 0.05	0.98 ± 0.05	0.68 ± 0.02	0.44 ± 0.02	0.97 ± 0.05	0.96 ± 0.05	1.23 ± 0.05	0.74 ± 0.02	0.49 ± 0.02	0.65 ± 0.02	0.79 ± 0.02
ΣMUFA		23.45 ± 1.04	24.24 ± 0.52	26.05 ± 0.72	33.64 ± 0.47	29.76 ± 1.12	18.60 ± 0.37	19.47 ± 0.77	24.89 ± 1.09	28.17 ± 1.17	28.40 ± 1.39	22.90 ± 0.89	25.37 ± 0.97	26.27 ± 0.47	26.74 ± 0.70
14.76	16:3n7	1.73 ± 0.07	3.20 ± 0.12	2.15 ± 0.10	1.58 ± 0.05	1.13 ± 0.02	4.63 ± 0.17	4.06 ± 4.13	1.39 ± 0.10	1.67 ± 0.07	2.27 ± 0.10	2.01 ± 0.07	2.25 ± 0.07	2.27 ± 0.10	2.88 ± 0.15
18.24	18:3n6	0.78 ± 0.05	1.26 ± 0.05	0.67 ± 0.02	0.93 ± 0.02	1.11 ± 0.05	0.66 ± 0.02	0.91 ± 0.02	0.89 ± 0.02	1.03 ± 0.05	1.95 ± 0.07	0.82 ± 0.05	0.80 ± 0.02	0.94 ± 0.05	0.90 ± 0.05
18.55	18:2n9	21.45 ± 0.67	25.670.70	24.64 ± 0.94	32.36 ± 0.40	31.01 ± 0.60	23.28 ± 0.77	22.64 ± 0.77	20.76 ± 0.97	21.22 ± 0.17	31.83 ± 0.84	27.62 ± 0.97	29.98 ± 0.32	31.95 ± 0.65	20.17 ± 1.19
21.81	20:3n8	0.58 ± 0.02	0.65 ± 0.02	0.81 ± 0.02	0.41 ± 0.02	0.39 ± 0.02	0.57 ± 0.02	0.41 ± 0.02	0.98 ± 0.05	0.77 ± 0.05	0.64 ± 0.02	0.74 ± 0.02	0.85 ± 0.02	0.64 ± 0.02	0.98 ± 0.05
ΣPUFA		24.53 ± 1.02	30.77 ± 0.89	28.26 ± 0.77	35.28 ± 0.77	33.63 ± 0.87	29.14 ± 2.73	28.02 ± 0.82	24.02 ± 1.09	24.68 ± 0.55	36.69 ± 0.50	31.19 ± 0.74	33.88 ± 0.97	35.79 ± 0.82	24.94 ± 0.50
34.06	10-nonadecanone	5.45 ± 0.32	0.09 ± 0.00	5.26 ± 0.15	5.52 ± 0.17	6.27 ± 0.07	9.91 ± 0.17	10.42 ± 0.17	6.91 ± 0.07	8.24 ± 0.12	6.93 ± 0.25	6.71 ± 0.30	11.28 ± 0.35	3.23 ± 0.12	10.57 ± 0.55
36.47	Stigmasterol	3.43 ± 0.20	2.00 ± 0.10	2.37 ± 0.07	1.37 ± 0.05	2.00 ± 0.10	2.20 ± 0.10	2.92 ± 0.07	3.24 ± 0.12	2.64 ± 0.17	1.50 ± 0.05	2.50 ± 0.10	1.89 ± 0.07	1.67 ± 0.05	1.78 ± 0.07
37.30	*β*-sitosterol	2.75 ± 0.07	3.71 ± 0.15	2.63 ± 0.12	1.93 ± 0.10	1.58 ± 0.07	1.88 ± 0.05	2.02 ± 0.07	1.72 ± 0.05	1.92 ± 0.07	2.29 ± 0.05	3.36 ± 0.07	2.28 ± 0.05	2.12 ± 0.07	2.47 ± 0.05
ΣOthers		11.63 ± 1.04	5.80 ± 0.15	10.27 ± 0.07	8.82 ± 0.05	9.85 ± 0.05	13.99 ± 0.62	15.36 ± 0.32	11.87 ± 0.47	12.80 ± 0.27	10.73 ± 0.52	12.56 ± 0.17	15.45 ± 0.40	7.03 ± 0.12	14.81 ± 0.72

^c^ RT: retention time.

**Table 3:** Characteristics of *Bupleurum scorzonerifolium* Willd. samples.

Sample Code	Voucher Number or Mark	Date of Collection	Yield of Essential Oil, *v*/*w* (%)	Yield of Lipids, *w*/*w* (%)	Location	Latitude Longitude	Attitude (m)
1	TZA-2014-1	14-07-2014	1.66	2.73	Surroundings of the Sotnikovo village, Ivolginsky district, Buryatia	N51°55.2′ E107°28.2′	490
2	TZA-2015-1	16-06-2015	0.30	2.97	Surroundings of the Sotnikovo village, Ivolginsky district, Buryatia	N51°55.2′ E107°28.2′	490
3	UUH017463	12-08-2017	0.62	3.48	Surroundings of the Sotnikovo village, Ivolginsky district, Buryatia	N51°55.2′ E107°28.2′	490
4	TZA-2014-2	15-07-2014	0.38	-	Surroundings of the Ivolga village, Ivolginsky district, Buryatia	N51°42.2′ E107°12.2′	645
5	TZA-2015-2	23-06-2015	0.80	4.88	Surroundings of the Zagustay arbor, Selenginsky district, Buryatia	N50°53.4′ E106°13.0′	572
6	TZA-2015-3	22-07-2015	0.18	3.62	Surroundings of the Georgievka village, Khorinsk district, Buryatia	N52°22.1′ E110°28.4′	769
7	UUH017680	22-08-2016	0.33	2.39	Surroundings of the Oninoborsk village, Khorinsk district, Buryatia	N52°13.6′ E109°57.1′	772
8	UUH017349	26-07-2016	0.31	9.38	Surroundings of the Shiringa village, Eravninsky district, Buryatia	N47°47.2′ E107°13.2′	926
9	TZA-2014-3	06-07-2014	0.32	8.57	Surroundings of the Nyzhniy Tsasuchey village, Ononsky district, Trans–Baikal region	N50°49.9′ E113°33.4′	811
10	TZA-2014-4	15-08-2014	0.31	8.77	Surroundings of the Bayan Ulaan Uul mountain, Khentii aimag	N47°20.2′ E109°07.2′	1327
11	TZA-2015-4	17-08-2015	0.51	5.81	Surroundings of the Bayan Ulaan Uul mountain, Khentii aimag	N47°20.2′ E109°07.2′	1327
12	TZA-2014-5	12-08-2014	0.38	5.62	Surroundings of the Berkh territory, Khentii aimag	N47°49.2′ E111°14.2′	1091
13	TZA-2015-5	06-08-2015	0.51	5.81	Surroundings of the Berkh territory, Khentii aimag	N47°49.2′ E111°14.2′	1091
14	TZA-2014-6	29-07-2014	0.33	5.81	In 20 km to northeast from the Berkh territory, Khentii aimag	N47°84.0′ E111°52.4′	1015
15	TZA-2014-7	23-07-2014	0.34	8.77	Surroundings of the Huh nuur Lake, Khentii aimag	N48°01.2′ E108°57.4′	1670
